# Novel mutation in RPGRIP1L gene causing Joubert syndrome: A case report

**DOI:** 10.1097/MD.0000000000035600

**Published:** 2023-11-24

**Authors:** Paola Andrea Duque-Cordoba, Lorena Diaz-Ordoñez, Juan David Gutierrez-Medina, Harry Pachajoa

**Affiliations:** a Centro de Investigaciones en Anomalías Congénitas y Enfermedades Raras (CIACER), Universidad Icesi, Cali, Colombia; b Departamento de Ciencias Básicas Médicas, Facultad de Salud, Universidad Icesi, Cali, Colombia; c Genetic Division, Fundación Valle del Lili, Cali, Colombia; d Centro de Investigaciones Clínicas, Fundación Valle del Lili, Cali, Colombia.

**Keywords:** case report, exome sequencing, Joubert syndrome, mutation, rare diseases, RPGRIP1 Like RPGRIP1L, sequence analysis

## Abstract

**Introduction:**

Joubert syndrome is a rare disease of genetic origin with autosomal recessive inheritance and extreme genetic heterogeneity with more than 40 causative genes. Joubert syndrome 7 is caused by mutations in the RPGRIP1L gene.

**Patient concerns:**

Our report describes a pediatric patient with clinical features compatible with JS type 7 such as hypotonia, developmental delay and aplasia of the cerebellar vermis.

**Diagnosis:**

The clinical features and the MRI of the head and neck which showed alterations at the level of the posterior fossa, with absence of the vermis and horizontal disposition of the cerebellar peduncles, were compatible with Joubert syndrome. Whole exome sequencing detected the variants RPGRIP1L (NM_015272.2) c.697A > T (p. Lys233Ter) and RPGRIP1L (NM_015272.2) c.3545 del (p.Pro1182LeufsTer25).

**Interventions:**

Resection was performed to correct the polydactyly. At age 2 years umbilical hernia, adenoid surgery and ventilatory tubes surgery were performed. Renal biopsy confirmed interstitial fibrosis and focally accentuated mild tubular atrophy with focal tubular hypertrophy, compatible with the clinical suspicion of Joubert syndrome. Congenital hip dislocation surgery was performed. The patient underwent surgery for correction of concomitant divergent strabismus and continued with glasses for astigmatism and hyperopia.

**Outcomes:**

Sanger sequencing confirmed the patient´s results and the father was found to be a carrier of RPGRIP1L (NM_015272.2) c.697A > T (p. Lys233Ter) and the mother and maternal grandmother as carriers of RPGRIP1L (NM_015272.2) c.3545del (p.Pro1182LeufsTer25). RPGRIP1L:c.3545del novel variant is a deletion which changes the reading frame, altering the RPGR1_C terminal domain and giving rise to an incomplete protein whose functions will be altered.

**Conclusion:**

This is the first genetically confirmed case of JS in Colombia, the first carrier of biallelic RPGRIP1L gene mutations with hip dislocation and incomplete glottic closure and the first report of the novel c.3545del likely pathogenic variant causing JS.

## 1. Introduction

Joubert syndrome (JS) is a rare multisystemic genetic disorder, first described in 1969 by Marie Joubert in a family where 4 of the children presented agenesis of the vermis and whose initial clinical findings were: hyperpnea, abnormal eye movement, intellectual disability and ataxia.^[1]^ Although JS prevalence has not been determined, different authors use a range between 1:80,000 and 1:100,000 considering cases described in Netherlands, China, Japan, Saudi Arabia, Switzerland, Germany, France and Morocco.^[2–4]^ JS prevalence in Latin America is unknown, however is estimated to be similar to the global prevalence. Specifically in Colombia, only 1 case has been reported of a 39-year-old adult diagnosed with JS, who presented intellectual disability, molar tooth sign (MTS) in magnetic resonance imaging (MRI) and sleep disorders such as lower limb movements, snoring, respiratory pauses and daytime sleepiness; clinical features that are uncommon in JS and that were previously described in a Japanese patient in which the related genetic mutation is not mentioned.^[5–7]^

JS is characterized by the neuro-radiological image known as MTS which corresponds to aplasia or hypoplasia of the cerebellar vermis, accompanied by thickened and reoriented superior peduncles,^[8]^ that in turn leads to other neurological signs such as neonatal hyperpnea, developmental delay, hypotonia, ataxia and variable intellectual disability.^[6]^ The multiorgan symptomatology is related to the mutated gene, which is why a clinical classification according to the main affectations was proposed.^[4]^

Mutations associated with JS are mainly related to a defect in the primary cilia, which includes JS in a group of disorders known as “ciliopathies.” The primary cilia are composed of a basal body, a transitional zone and the axoneme, cell membrane structures responsible for signal transduction. Mutations in the RPGRIP1L gene can affect various areas of the cilium, interrupting functions such as the entry and exit of ciliary proteins through intraflagellar transport, or Hedgehog signaling, which participates in the development and growth of various organs and tissues such as renal, hepatic and neural tissue.^[9–11]^

To date, mutations associated with JS have been found in 40 genes: 39 genes with autosomal recessive inheritance and 1 X-linked. These variations have generated a genetic classification where the number of the JS type is associated with each mutated gene.^[12]^ Joubert syndrome 7 is caused by mutations in the RPGRIP1L gene which is located on chromosome 16q12.2 and encodes for the fantom protein, responsible for several functions at the cellular level such as negative regulation of cell signaling, embryonic development (craniofacial and limb), programmed cell death and regulation of the proteosomal activity of primary cilia.^[4]^ According to NCBI (https://www.ncbi.nlm.nih.gov/), isoform A, the longest of the 6 isoforms of the fantom protein, is composed of 1315 amino acids and contains 5 domains which interact lysosomal proteins 1 and 2 and the X-linked retinitis pigmentosa GTPase regulator. Here we describe the case of a patient with clinical characteristics compatible with Joubert syndrome and a novel deleterious mutation in the RPGRIP1L gene that had not been reported in the literature before.

## 2. Ethics approval and consent to participate

All research was conducted according to the Declaration of Helsinki and the research protocol was registered with the number 1504 upon approval from the IRB Biomedical Research Ethics Committee of the Fundacion Valle del Lili. The patient and patient family provided their written informed consent to participate in this study.

## 3. Patient consent for publication

The patient and patient family provided their written informed consent for the publication of this study. Written informed consent was obtained from the patient and parents for the publication of the case details and accompanying images.

## 4. Case presentation

A 16-year-old Colombian male patient of non-consanguineous parents, the pregnancy did not present complications and no significant findings were found in prenatal controls. Was delivered at 40 weeks of pregnancy with a weight of 3500 grams and height of 54 cm, APGAR 8, postaxial polydactyly was observed in the metacarpal of the left hand, prominent forehead and an increase in the diameter of the head, for which a presumptive diagnosis of hydrocephalus was made.

At 8 months of age, when resection was performed to correct the polydactyly, spina bifida occulta in S1 and scoliosis in the left dorsolumbar junction were detected by radiography. A computerized tomography scan showed ventriculomegaly and hydrocephalus without progression, physical and occupational therapy was performed to improve axial-trunk-cervical tone and hypotonia. The auditory evoked potentials were OD: 20 db and OI: 30 db, the Impedanciometry detected OD curve type C suggestive of tubal dysfunction, OI: curve type B suggestive of middle ear pathology, with absent reflexes. Chromosomopathy and Soto syndrome or cerebral gigantism were ruled out due to the normal karyotype 46 XY. At age 1 year hydrocephalus was ruled out and Robinow syndrome was suspected. A simple brain scan was performed where prominent lateral ventricles were found, and metabolic screening was also performed, which was negative.

At age 2 years umbilical hernia and ventilatory tubes surgery were performed, which helped to improve reflexes and balance. Additionally, an MRI of the head and neck showed alterations at the level of the posterior fossa, with absence of the vermis and horizontal disposition of the cerebellar peduncles, characteristics that are compatible with Joubert syndrome. At the age of 5 years a second surgery of ventilatory tubes and adenoid surgery was performed, which helped to correct problems of severe night snoring and frequent rhinorrhea.

At the age of 8 years, the WISC-RM Children Intelligence Test was performed to obtain the IQ, with a total score of 78 (mild cognitive disability); the verbal comprehension score was 71 (borderline range) and the manipulative score was 73 (borderline range). At age 11 years a renal biopsy was performed due to the suspicion of a possibility of Juvenile Nephronophthisis related to JS, interstitial fibrosis and focally accentuated mild tubular atrophy with focal tubular hypertrophy were found, compatible with the clinical suspicion.

**Figure 1.**
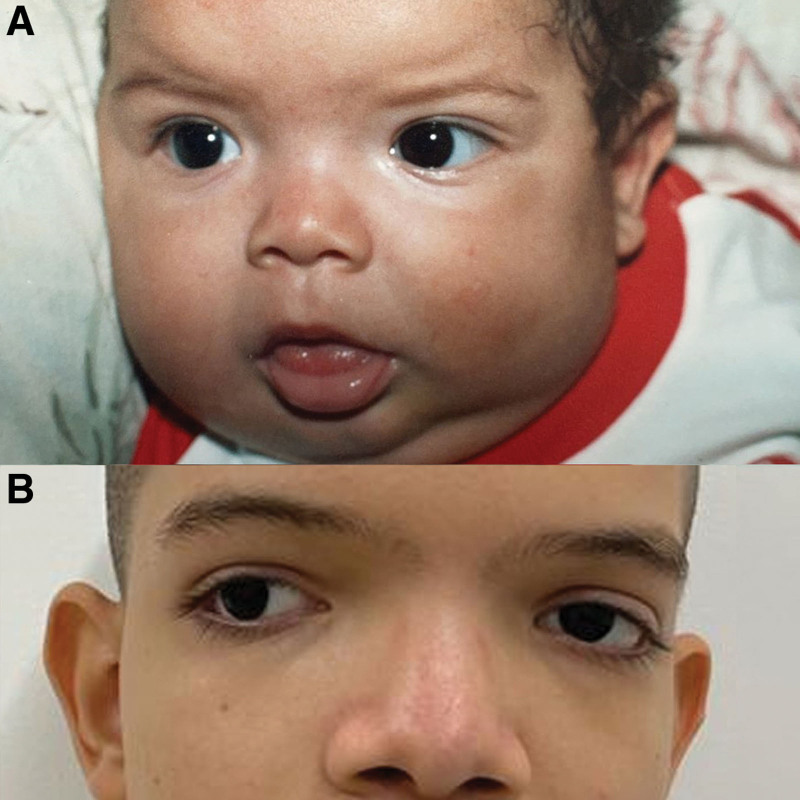
Physical characteristics (A) Photo of the patient at age 5 months showing broad nasal bridge and prominent forehead (B) Photo of the patient at age 16 years, showing strabismus, ocular hypertelorism and broad nasal bridge.

**Figure 2.**
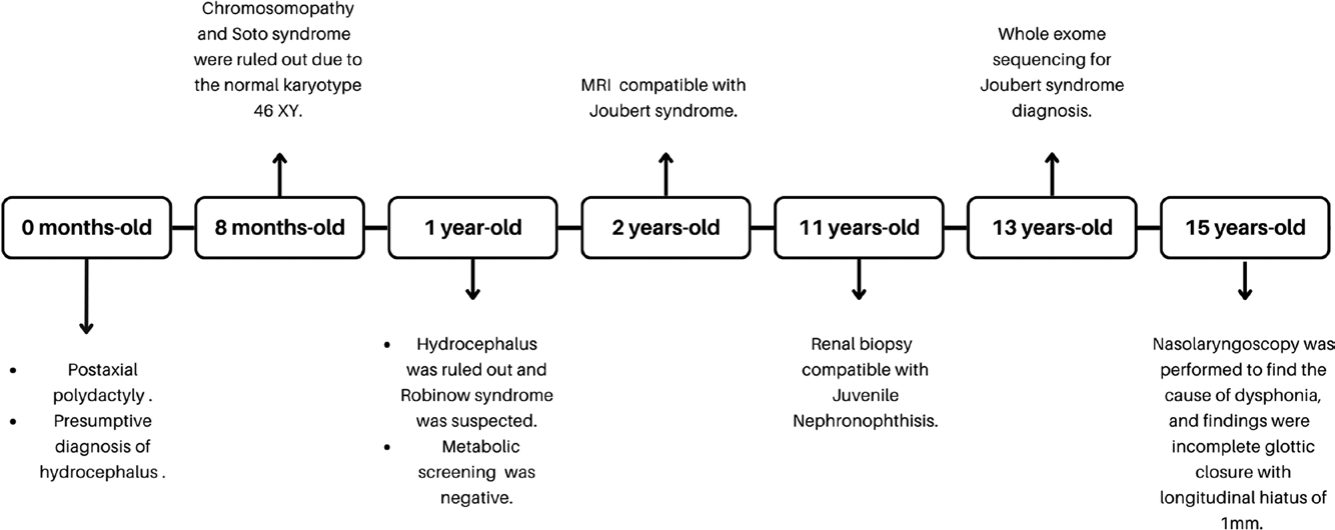
Timeline of clinical events.

At age 15 years a congenital hip dislocation was detected by an X-ray, for which corrective surgery was performed. In the same year, a nasolaryngoscopy was performed to find the cause of dysphonia, and findings were made of granular pharyngeal walls; arytenoids and interarytenoid fold, with edema and mild erythema; and mobile vocal cords, with incomplete glottic closure with longitudinal hiatus of 1mm. The patient underwent surgery for correction of concomitant divergent strabismus and continued with glasses for astigmatism and hyperopia. Physical examination revealed dolichocephaly, easy asymmetry, telecanthus, ocular hypertelorism, wide nasal bridge and high palate (Fig. 1). All surgical procedures performed on the patient were tolerated, and there were no adverse or fortuitous events. In addition, since the COVID-19 pandemic began, the patient has not undergone new interventions, and he only continues with routine checkups. Clinival events are presented in Figure 2.

Whole exome sequencing (WES) was performed at PerkinElmer Genomics on genomic DNA using the Agilent v6CREv2 targeted sequence capture method to enrich the exome. Direct sequencing of the amplified captured regions was performed using 2 × 100bp reads on Illumina next generation sequencing (NGS) systems. Coverage of 99.3% of the target bases of the disease-causing gene was obtained, and 99.1% of the exons. An average coverage of 139.2X was obtained considering that a base has sufficient coverage at 20X and an exon is considered completely covered if all coding bases plus 3 nucleotides of the intronic sequence on each side are covered at 20X or more. This exome detected the variants RPGRIP1L (NM_015272.2) c.697A > T (p. Lys233Ter) and RPGRIP1L (NM_015272.2) c.3545 del (p.Pro1182LeufsTer25), the latter has not been reported in databases such as Clinvar, ExAC, 1000 genomes and HGMD and is classified as a probably pathogenic variant according to the recommendations of the American College of Medical Genetics and Genomics; Polyphen- 2 presents a score of 0.987 (Probably harmful) in HumDIV and a score of 0.645 (possibly harmful) in HumVAR.

**Figure 3.**
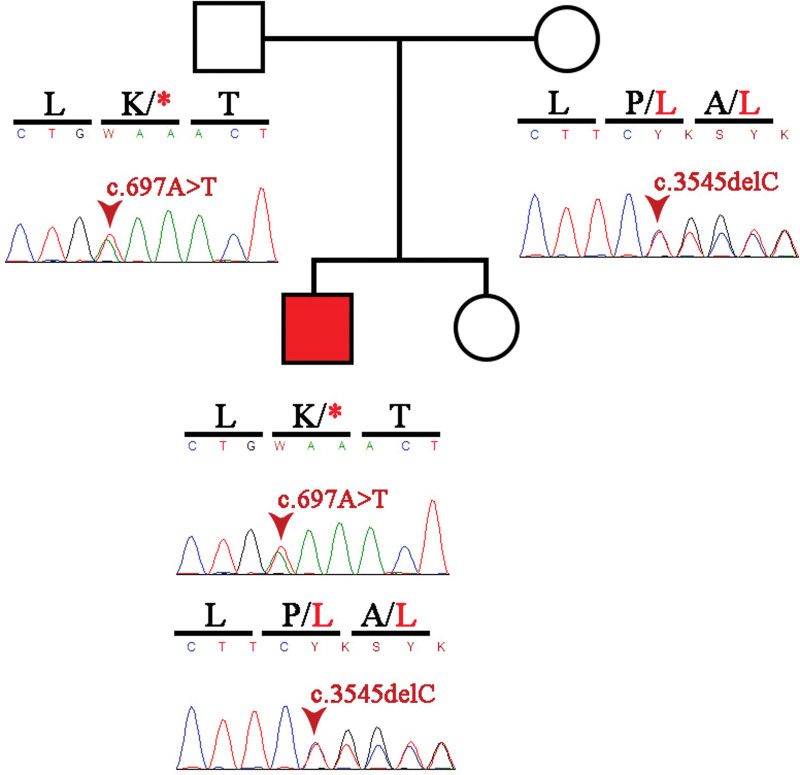
Genogram and Sanger sequencing electropherograms (A) and (B) patient, (C) father and (D) mother. Both RPGRIP1L autosomal recessive mutations are observed to be inherited.

In order to confirm the variants, blood samples were collected from the patient and family members in 4mL EDTA tubes and DNA extraction was performed using the QIAamp DNA Mini Kit (QIAGEN, Germany) following the manufacturer protocol. polymerase chain reaction (PCR) primers for exons 6 and 24 of the RPGRIP1L gene were designed through Primer3 software (https://bioinfo.ut.ee/primer3-0.4.0): exon 6 forward primer 5’-ACAAGTTCTTACAGAGGCTCTT-3’ and reverse primer 5’- AGCAGTTAGTAGGTCGAGCA-3’; and exon 24 forward primer 5’- TGCATTCAAGAAAAGAAGCCCA-3’ and reverse primer 5’- TCTCTCTGTTCTTTCCACTTCTT-3’. PCR was performed in a final volume of 25 uL: 12.45 uL of water, 5 uL of Flexi Buffer, 4 uL of MgCl2, 1 uL of dNTPs, 0.625 uL of each primer and 0.3 uL of GoTaq G2 Flexi DNA Polymerase (Promega, USA). PCR amplification was performed using the following cycle conditions: 95 °C for 5 minutes; 35 cycles consisting of 95 °C for 30 seconds, specific annealing temperature (62°C for exon 6 primers and 57°C for exon 24 primers) for each pair of primers for 45 seconds, 72°C for 8 minutes and a final extension at 72 °C for 8 minutes. PCR products (329bp for exon 6 and 389 for exon 24) were analyzed on a 1% agarose gel and then purified with the QIAquick PCR Purification kit (QIAGEN, Germany) and used for sanger sequencing using the BigDye Terminator v.3.1 (Applied Biosystems, Foster City, USA) following the manufacturer instructions. The sanger sequencing products were then purified with the BigDye Xterminator Purification Kit (Applied Biosystems, Thermo Fisher Scientific, USA). For the sequencing of exons 6 and 24, reverse and forward primer were used, respectively, using the ABI 3500 (Applied Biosystems, Thermo Fisher Scientific, Waltham, MA, USA). The patient results were confirmed and the father was found to be a carrier of RPGRIP1L (NM_015272.2) c.697A > T (p. Lys233Ter) and the mother and maternal grandmother as carriers of RPGRIP1L (NM_015272.2) c.3545del (p.Pro1182LeufsTer25) (Fig. 3).

## 5. Discussion

JS is mainly diagnosed with MRI where MTS is evidenced, in addition to other clinical manifestations such as hypotonia, ataxia, developmental delay and intellectual disability. There are 40 genes associated with this pathology, which are involved in the function and structure of the primary cilia. The type of JS will depend on the clinical features and the associated genetic mutations.^[12]^

Our patient is compound heterozygous for mutations in the RPGRIP1L gene. We found the RPGRIP1L:c.697A > T (p. Lys233Ter) variant located in exon 6, which codes for a stop codon at amino acid 233 affecting the structure of the protein upstream of the Fez1 domain, giving rise to a truncated protein that can alter the functionality of RPGRIP1L. On the other hand, the RPGRIP1L:c.3545del variant (p.Pro1182LeufsTer25) is a deletion which changes the reading frame, turning Proline 1182 into a Leucine and introducing a stop codon 25 amino acids later, altering the RPGR1_C terminal domain, and giving rise to an incomplete protein whose functions will also be altered. Both mutations classify our patient as SJ type 7 and his phenotype is justified by an impairment in the interaction with Nephrocystin-4, a protein associated with nephronophthisis.^[13]^

RPGRIP1L mutations present a renal phenotype in 72% of cases. Our patient presents with stage 3a chronic kidney disease, which is consistent with the reported literature.^[2,3,9]^ Intellectual disability (68%), developmental delay (60%) and cerebral ataxia (60%) are common clinical features in patients with RPGRIP1L mutations (see Supplementary Table 1, http://links.lww.com/MD/K804, Supplemental Content, which illustrates the patient clinical features in different articles regarding RPGRIP1L mutations causing JS), which are related to the high expression of RPGRIP1L in the fetal brain, where is assumed to play an important role in the embryonic development of the fetus.^[14]^ Oculomotor alterations are present in 48% of the cases, although the type of alteration varies according to the patient. Strabismus, although less frequent, was present in 16% of the cases^[15–21]^ (Supplementary Table 1, http://links.lww.com/MD/K804).

Polydactyly was found in 20% of the cases reviewed, including ours, but it should be noted that it was absent in a heterozygous patient who presented one of the mutations in our case (c.697A > T). Hearing problems were only present in our case, as well as congenital dislocation (Supplementary Table 1, http://links.lww.com/MD/K804). Scoliosis was present in 3 other cases and there was only 1 case of hernia. It is worth bearing in mind that the oropharyngeal malformation of incomplete glottic closure was only present in our case and is not characteristic of RPGRIP1L, since the gene mainly associated with oropharyngeal malformation is TMEM216.

Kroes et al^[22]^ conducted a study with a cohort of 51 patients with JS in Northern Europe, where only 1% had mutations in the RPGRIP1L gene. Arts et al^[9]^ conducted a study with a cohort of 68 patients with Joubert syndrome, where only 5% corresponded to mutations in the RPGRIP1L gene. In Colombia, the prevalence of JS and the genetic variants in this article is unknown, however it is estimated to be similar to that of the rest of the world. It is recommended to give greater importance to genetic analysis as a tool for the characterization and confirmation of JS cases, in order to perform a more focused control and treatment.

## 6. Conclusion

RPGRIP1L: c.3545del is reported as a new JS causing variant, which in this case has been inherited through the maternal line. Most of our patient symptoms are characteristic of RPGRIP1L disease (Joubert syndrome 7) with the exception of hip dislocation and incomplete glottic closure. Clinical suspicion and primary diagnoses were made based on clinical features. The importance of genetic diagnosis is emphasized since in Colombia there are no prevalence or molecular diagnosis data regarding JS.

## 7. Limitations and strengths of the study

We described thoroughly the second case of Joubert syndrome reported in Colombia and the first to be genetically confirmed through Next Generation Sequencing and Sanger sequencing. We provided bioinformatic explanation of the pathogenic mutations and provided a comparison between the available literature regarding RPGRIP1L mutations and the clinical manifestations of our patient. One of the limitations of our study is that the molecular mechanism underlying the pathogenesis of Joubert syndrome caused by the RPGRIP1L:c.3545del mutation need to be further investigated and described.

## Author contributions

**Conceptualization:** Harry Pachajoa.

**Data curation:** Paola Andrea Duque, Juan David Gutierrez-Medina.

**Investigation:** Paola Andrea Duque.

**Methodology:** Paola Andrea Duque, Lorena Diaz-Ordoñez, Juan David Gutierrez-Medina.

**Project administration:** Harry Pachajoa.

**Resources:** Harry Pachajoa.

**Supervision:** Lorena Diaz-Ordoñez, Harry Pachajoa.

**Validation:** Paola Andrea Duque, Lorena Diaz-Ordoñez, Juan David Gutierrez-Medina.

**Writing – original draft:** Paola Andrea Duque, Lorena Diaz-Ordoñez, Juan David Gutierrez-Medina.

**Writing – review & editing:** Paola Andrea Duque, Lorena Diaz-Ordoñez, Juan David Gutierrez-Medina.

## Supplementary Material
