# Supplementary material for: Novel mutation in RPGRIP1L gene causing Joubert syndrome: A case report
Source: Medicine (Baltimore). 2023 Nov 24;102(47):e35600. doi: 10.1097/MD.0000000000035600 (PMC10681469; doi:10.1097/MD.0000000000035600)
Supplement: Supplementary file 1 [file medi-102-e35600-s001.pdf]

Supplementary Table 1. Comparison of the patient's clinical features in different articles regarding RPGRIP1L mutations causing Joubert syndrome.

Article	This case	Wolf et al (2007)[2]					Delous et al (2007)[3]						Arts et al (2007)[9]			Doherty et al (2010)[16]	Takagi et al (2021)[17]	Tian et al (2021)[18]	Radha et al (2020)[19]		Kroes et al (2016)[15]	Chaki et al (2011)[20]	Halbritter et al (2013)[21]	Alazami et al (2012)[22]	Brancati et al (2008)[14]	Total	Percentage
**Mutation**	697A>T	1843A>C	1897T>C	IVS23-2A>G	1177G>A	1843A>C	697A>T	757C>T	3686delA	2083G>C	2083G>C	2030C>T	2305-1G>A	2050C>T	1721delA	2413C>T/1975T>C	86-2A>G	2018G>A	787C>T	346C>T	843A>C	c.2450A>G	c.1700-1A>G	c.1649A>G	c.2268_2269delA	25	
	3545del						1843A>C	1843A>C						1843A>C			231-2A>G		3811_3812ins		712del						
**Country**	Colombia	USA	Germany	Germany	Germany	Switzerland	France	France	France	France	France	Comoro Islands	Turkey	Northern Europe	Turkey	NR	Japan	China	NR	NR	NR	NR	USA	Saudi Arabia	NR		
**Molar Tooth**	YES	NR	YES	YES	NR	YES	NR	YES	YES	YES	YES	NR	YES	YES	YES	YES	NR	YES	NR	NR	NR	NR	NR	YES	YES	15	60%
**Intellectual disability**	YES	YES	YES	YES	YES	YES	YES	YES	YES	YES	NR	YES	YES	NR	YES	NO	YES	NR	NO	NR	NO	YES	NR	YES	YES	17	68%
**Developmental delay**	YES	NR	NR	NR	NR	NR	NR	NR	NR	NR	NR	NR	YES	YES	YES	YES	YES	NR	YES	NR	YES	NR	NR	YES	YES	10	40%
**Renal phenotype**	YES	YES	YES	YES	YES	YES	YES	YES	YES	YES	YES	YES	No	NR	YES	YES	YES	YES	NR	NR	YES	YES	YES	NR	YES	18	72%
**Polydactyly**	YES	NR	NR	NR	NR	NR	NR	NR	NR	NR	NR	NR	NR	YES	NR	NR	NO	NR	NR	NR	YES	NR	YES	NR	YES	5	20%
**Hearing problems**	YES	NR	NR	NR	NR	NR	NR	NR	NR	NR	NR	NR	NR	NR	NR	NR	NO	NR	NR	NR	NR	NR	NR	NR	NR	1	4%
**Hepatic impairment**	NR	NR	NR	YES	YES	NR	NR	NR	NR	NR	NR	NR	NR	NR	NR	YES	YES	NR	YES	NR	NR	NR	NR	NR	NR	5	20%
**Cerebral ataxia**	YES	NR	YES	YES	NR	YES	YES	YES	YES	YES	YES	YES	YES	NR	YES	YES	NO	NR	NR	NR	NR	NR	NR	YES	YES	15	60%
**Retinal degeneration**	NR	NR	NR	NR	NR	NR	NR	NR	NR	NR	NR	NR	NR	NR	NR	NR	YES	NR	NR	YES	NR	NR	NR	NR	NR	2	8%
**Strabismus**	YES	NR	NR	NR	NR	NR	NR	NR	NR	NR	NR	NR	NR	YES	NR	NR	NR	NR	NR	NR	NR	NR	NR	YES	YES	4	16%
**Oculomotor involvement**	YES	NR	NR	NR	NR	SÏ	YES	YES	YES	YES	YES	YES	YES	YES	YES	YES	NR	NR	NR	NR	NR	YES	NR	YES	YES	12	48%
**Others**	Prominent forehead, scoliosis, oropharyngeal malformation, congenital hip dislocation, hernia.	NR	NR	Neonatal tachypnea	Coloboma	NR	Genu valgum	Scoliosis	Partial GH deficiency	Scoliosis	Scoliosis	Precocious puberty	NR	NR	NR	Abnormal breathing	Hidrocephaly	NR	NR	NR	Apnea	NR	Microcephaly	Prominent forehead	Club foot, inginal hernia, apnea		

NR: Not reported (This information is not discussed in the article)
